# Characterization of Laminar Zones in the Mid-Gestation Primate Brain with Magnetic Resonance Imaging and Histological Methods

**DOI:** 10.3389/fnana.2015.00147

**Published:** 2015-11-24

**Authors:** Xiaojie Wang, David R. Pettersson, Colin Studholme, Christopher D. Kroenke

**Affiliations:** ^1^Division of Neuroscience, Oregon National Primate Research Center, Oregon Health & Science UniversityBeaverton, OR, USA; ^2^Department of Radiology, Oregon Health & Science UniversityPortland, OR, USA; ^3^Biomedical Image Computing Group, Departments of Pediatrics, Bioengineering, and Radiology, University of WashingtonSeattle, WA, USA; ^4^Advanced Imaging Research Center and Department of Behavioral Neuroscience, Oregon Health & Science UniversityPortland, OR, USA

**Keywords:** rhesus macaque, fetal MRI, brain development, subventricular zone, cortical plate, subplate, MRI validation, diffusion MRI

## Abstract

Distinct populations of progenitor and postmitotic neural and glial cells are stratified in the fetal primate brain across developmentally transient tissue zones between the ventricular and pial surfaces. These zones were originally identified by light microscopy. However, it has subsequently been shown that various forms of magnetic resonance image (MRI) contrast can be used to distinguish layers of developing neural tissue in *ex vivo*, as well as *in vivo* (including *in utero*) conditions. Here we compare mid-gestation rhesus macaque tissue zones identified using histological techniques to *ex vivo* as well as *in utero* MRI performed on the same brains. These data are compared to mid-gestation fetal human brain MRI results, obtained *in utero*. We observe strong similarity between MRI contrast *in vivo* and post mortem, which facilitates interpretation of *in utero* images based on the histological characterization performed here. Additionally, we observe differential correspondence between the various forms of *ex vivo* MRI contrast and microscopy data, with maps of the water apparent diffusion coefficient providing the closest match to histologically-identified lamina of the nonhuman primate brain. Examination of histology and post mortem MRI helps to provide a better understanding of cytoarchitectrual characteristics that give rise to *in utero* MRI contrast.

## Introduction

The mid-gestation primate brain is organized into layers, with those proximal to the surface of the lateral ventricles containing progenitor cell populations, and layers proximal to the pial surface containing postmitotic neurons and glial cells (Bayer and Altman, 2005; Bystron et al., 2008; Cunningham et al., 2013). In gyroencephalic species such as humans and old world monkeys, multiple distinct layers that are not apparent in MRI data collected from mammals with smaller brains, can be resolved by MRI at this developmental stage (Kriegstein et al., 2006; Sizonenko et al., 2007; Huang et al., 2008; Barnette et al., 2009). Methods for classifying these bands differ between researchers. However, descriptions of 8–10 layers between the ventricular and pial surface has been common to most recent studies of primate species (Altman and Bayer, 2002; Kostovic et al., 2002; Smart et al., 2002; Bystron et al., 2008). Increasingly, the importance of progenitor cell populations residing within subventricular zones is being recognized in contributing to the number of cerebral cortical neurons in the mature brain (Hansen et al., 2010) and potentially to the shape of the folded cortex (Reillo et al., 2011). In primate brain at this developmental stage, more superficial layers contain migrating postmitotic cells, as well as axons that will contribute to white matter structures in the mature brain. Experimental strategies that can be used to quantitatively characterize the physical properties (e.g., size and cellular organization) of these tissue zones throughout development are therefore of potential utility for monitoring normal and pathological brain development.

Magnetic resonance imaging (MRI) has long been used as a clinical tool for characterizing human fetal brain development (Glenn and Barkovich, 2006). However, until recently, challenges associated with fetal brain motion have limited the choice of MRI contrast mechanism, and achievable image resolution. Strategies for retrospective motion correction have ameliorated these limitations (Studholme, 2011) and have enabled high resolution 3D reconstructions of T_2_-weighted images throughout the second half of gestation (Kim et al., 2011; Scott et al., 2011), as well as whole-brain diffusion tensor imaging (DTI) measurements on fetal brain (Fogtmann et al., 2014). Three tissue zones, termed the germinal matrix, the subplate (SP), and the cortical plate (CP), are typically resolved in *in utero* MRI studies (Kim et al., 2011). Post mortem MRI, allowing for lengthy scan time, affords the opportunity to obtain higher image resolution than achievable with *in utero* imaging measurements. (Gupta et al., 2005; Kroenke et al., 2005; Huang et al., 2006; Xu et al., 2014). Although MRI of post mortem tissue has provided evidence that additional tissue zones can be resolved with increased image resolution and signal to noise ratio (Kostovic et al., 2002; Kroenke et al., 2005, 2006; Huang et al., 2009; Zhang et al., 2011a,b; Kolasinski et al., 2013; Xu et al., 2014), the specific association between tissue zones defined using histological methods and MRI-based contrast patterns have not been established.

The goal for this study was to assign lamina identified by light microscopy in the mid-gestation rhesus macaque brain specifically to zones observable with MRI. Further, in order to determine the degree of correspondence to results observed in humans, comparisons are made between human and nonhuman primate MRI. Previously, Smart et al. (2002) classified the lamina identified in cresyl-violet stained parietal and occipital lobe tissue of the cynomolgus macaque. The banded structure across the cerebral wall differs between rostral and caudal brain regions (Altman and Bayer, 2002; Martínez-Cerdeño et al., 2012), and therefore the current study focuses on the same region characterized by Smart and co-workers. We demonstrate that the previously developed classification scheme can be applied to the parietal and occipital lobes of three rhesus brains at 90 days gestational age (G90, of a 165 day gestational term), which corresponds to ~23 weeks post-conception in humans (http://www.translatingtime.org) (Workman et al., 2013). We utilize *in utero* MRI, post mortem MRI, and light microscopic images of the same brains to establish the correspondence between zones identified by microscopy and those observed by MRI. Immunohistochemical analyses of radial glial cells and neural axons are also used to guide the interpretation of water diffusion anisotropy measurements performed on this tissue. Based on similarities between our findings and post-mortem MRI studies of the developing human brain, comparisons are made with classification systems described for human brains.

## Methods

### *In utero* MRI acquisition and image reconstruction

Human subjects provided written, informed consent to participate in this study, and all procedures involving human subjects were approved by the Oregon Health and Science University Institutional Review Board. A 23-week pregnant woman was imaged using a 1.5 T whole body MR scanner (Ingenia; Philips Healthcare, Netherlands). A dStream posterior built-in coil and a dSteam anterior torso radiofrequency (RF) coil (Philips Healthcare, Netherlands) were used for signal excitation and reception, respectively. Due to fetal head motion, it is often not possible to acquire a high-resolution 3D volume of the entire brain without implementing procedures such as retrospective motion correction (Rousseau et al., 2006). Therefore, the human imaging performed here follows the current standard clinical practice to acquire a selected series of 2D image slices. A 2D Single-Shot turbo spin-echo (TSE) pulse sequence was used to acquire axial T_2_-weighted images with the following parameters: recycle time (TR) = 1000 ms, echo time (TE) = 110 ms, Sensitivity encoding factor (SENSE, a parallel imaging acceleration parameter) = 3.6, halfscan factor = 0.64, TSE factor = 109, in plane resolution of 1 × 1 mm, and slice thickness of 3 mm.

All procedures involving nonhuman primate research subjects were approved by the Institutional Animal Care and Use Committee of the Oregon National Primate Research Center (ONPRC). The ONPRC abides by the Animal Welfare Act and regulations enforced by the U.S. Department of Agriculture, the Public Health Service Policy on Humane Care and Use of Laboratory Animals, in accordance with the U.S. National Institutes of Health Guide for the Care and Use of Laboratory Animals. Three pregnant rhesus macaques underwent anatomical examinations at G90 using a Siemens 3T Tim Trio system equipped with a 15-element human knee RF coil (QED, Cleveland, OH). Anesthesia was induced using 10 mg/kg ketamine and maintained using a 1.5% isoflurane–oxygen mixture during the imaging. A tri-plane localizer with half-Fourier acquisition single-shot turbo spin-echo (HASTE)-acquired T_2_-weighted image was used to determine fetal head position. A 2D TSE sequence was used to acquire T_2_-weighted images with the following parameters: TR/TE = 5000/97 ms, generalized autocalibrating partial parallel acquisition (GRAPPA) factor = 2, and TSE factor = 27. As described previously (Fogtmann et al., 2014), multiple contiguous 2D image stacks, with in-plane resolutions of 0.67 × 0.67 mm and thicknesses of 1 mm, were acquired along the maternal axial, sagittal, and coronal axes to facilitate the reconstruction of a 3D volume with isotropic resolution. For an additional pregnant rhesus macaque at G85, diffusion MRI data, in addition to T_2_-weighted images, were acquired. A diffusion-weighted, 2D spin-echo based EPI sequence was used to acquire one image volume with *b* = 0 (the “b0” image), and 20 diffusion-weighted volumes with *b* = 500 s/mm^2^. Other acquisition parameters were: TR/TE = 5000/93 ms, GRAPPA factor = 2, EPI factor = 78, and echo spacing = 1.09 ms. As with the T_2_-weighted images, three sets of b0 and diffusion weighted image stacks were acquired along the maternal axial, sagittal, and coronal axes. For the diffusion-weighted data, the in-plane resolution was 1.13 × 1.13 mm and the slice thickness was 3 mm. In order to compensate for the relatively poor through-plane resolution for the diffusion data, three sets of diffusion-weighted image stacks were acquired along each axis, offset from one another by 1 mm (Fogtmann et al., 2014).

For T_2_-weighted image stacks, 3D slice position and orientation correction with respect to fetal brain anatomy was carried out using the SLIMMER procedure (Kim et al., 2011). This incorporates the Slice Intersection Motion Correction (SIMC) algorithm and iterative bias field inconsistency correction to account for subtle changes in signal that occur when the fetal head moves in relation to the coils, and allows improved delineation of tissue contrast. The final 3D volume was reconstructed using an iterative deconvolution of the slice profiles in the orthogonal slice planes (Fogtmann et al., 2012, 2014) with 0.5 mm isotropic resolution. Reconstruction of DTI data was accomplished using the approach described in Fogtmann et al. (2014). Briefly, four major steps were involved: (1) a slice-to-volume alignment (Rousseau et al., 2006) was performed to generate a high-resolution b0 volume; (2) motion-estimates of the diffusion-weighted images were obtained using previously described procedures (Oubel et al., 2012) to register each slice in the diffusion-weighted data set to a common 3D volume; (3) the b0 volume, motion estimates, and DWI slices were then combined on a 0.75 mm isotropic 3D lattice to compute diffusion tensor parameters at each lattice point following procedures described in Gholipour et al. (2010); (4) The apparent diffusion coefficient [ADC = (λ_1_ + λ_2_ + λ_3_)/3] and fractional anisotropy [FA, defined in Basser and Pierpaoli (1996)] were calculated from the diffusion tensor results obtained in step 3.

### *Ex vivo* MRI acquisition and DTI analyses

Following Cesarean section, the three rhesus macaque fetuses imaged at G90 were euthanized with overdose of pentobarbital, and the brains were perfusion fixed with 4% paraformaldehyde (PFA), and then immersed in 4% PFA for 24 h before being transferred to phosphate-buffered saline (PBS). Immediately prior to imaging, the brains were transferred to Fluorinert Electronic Liquid FC-77 (3M, St. Paul, MN) and returned to PBS following MRI procedures. A custom Helmholtz coil (5 cm diameter, 5 cm length) was used for radiofrequency transmission and reception. Experiments were performed on an 11.7 T small-animal MRI system interfaced with 9 cm inner diameter magnetic field gradient coil diameter magnetic field gradient coil (Bruker, Rheinstetten, Germany). A multi-slice spin-echo pulse sequence (TR/TE = 15 s/30 ms), incorporating a Stejskal–Tanner diffusion sensitization gradient pair was used to acquire diffusion MRI data at an isotropic resolution of 0.3 mm. A 25-direction, icosahedral sampling scheme (Batchelor et al., 2003) was utilized for all experiments with 3 b0 images, and diffusion weighted images with a *b*-value of 2500 s/mm^2^. Standard procedures were followed to calculate eigenvalues (λ_1_, λ_2_, and λ_3_, listed from smallest to largest) and eigenvectors (V_1_, V_2_, and V_3_). DTI indices such as FA were calculated from the eigenvalues for each voxel. The signal intensity at a *b*-value of 0 was similarly estimated for each voxel, and the result served as the post mortem T_2_-weighted image.

### Histological analyses

Following *ex vivo* MRI examination, hemispheres from each of the G90 brains were cryo-protected in 15% and then 30% (w/v) sucrose-PBS solution before being frozen-sectioned in the axial plane at 80 μm thickness using a Zeiss-Microm sliding microtome (Dublin, California). For Nissl staining, sections were mounted onto 2% (w/v) gelatin subbed slides and processed following standard procedures (Paul et al., 2008). For immunohistochemistry, sections were stained in a free-floating fashion in 24-well cell culture plates. Briefly, each section was washed in PBS-Triton X-100 (0.1%, v/v) solution and then blocked with 5% (v/v) goat serum before being incubated with an anti-neurofilament marker SMI312 (1:1000, BioLegend Inc., San Diego, CA, USA) or monoclonal anti-vimentin antibody (1:40, Sigma-Aldrich, Saint Louis, MO, USA) in 4°C for 48 h. After washing in PBS-Triton X-100 solution, the sections were then incubated with secondary antibody conjugated with Alexa Fluor® 488 (1:500, Life Technologies, Grand Island, NY) for 2 h at room temperature. Before being mounted on gelatin subbed slides and sealed with cover slip, the sections were counter-stained with DAPI (Sigma-Aldrich, Saint Louis, MO, USA). A Leica SP5 ABOS confocal laser scan microscope (Leica Microsystems, Wetzlar, Germany) was used to acquire image stacks of SMI312- and vimentin- stained tissue sections. All images were collected using a 40x oil immersion objective at an in-plane resolution of (0.76 μm)^2^ and z step distance of 0.76 μm. Maximum projections of all image stacks were generated for display of vimentin-positive radial glial cells and SMI312-positive axons within regions of interest. A Zeiss Axioplan system (Carl Zeiss, Jena, Germany) interfaced with Stereo Investigator (MBF Bioscience, Williston, VT, USA) was used to acquire 2D (single z depth) montage photos of Nissl stained sections with an in-plane resolution of (2.1 μm)^2^ using a 5X objective.

## Results

**Figure 1:**
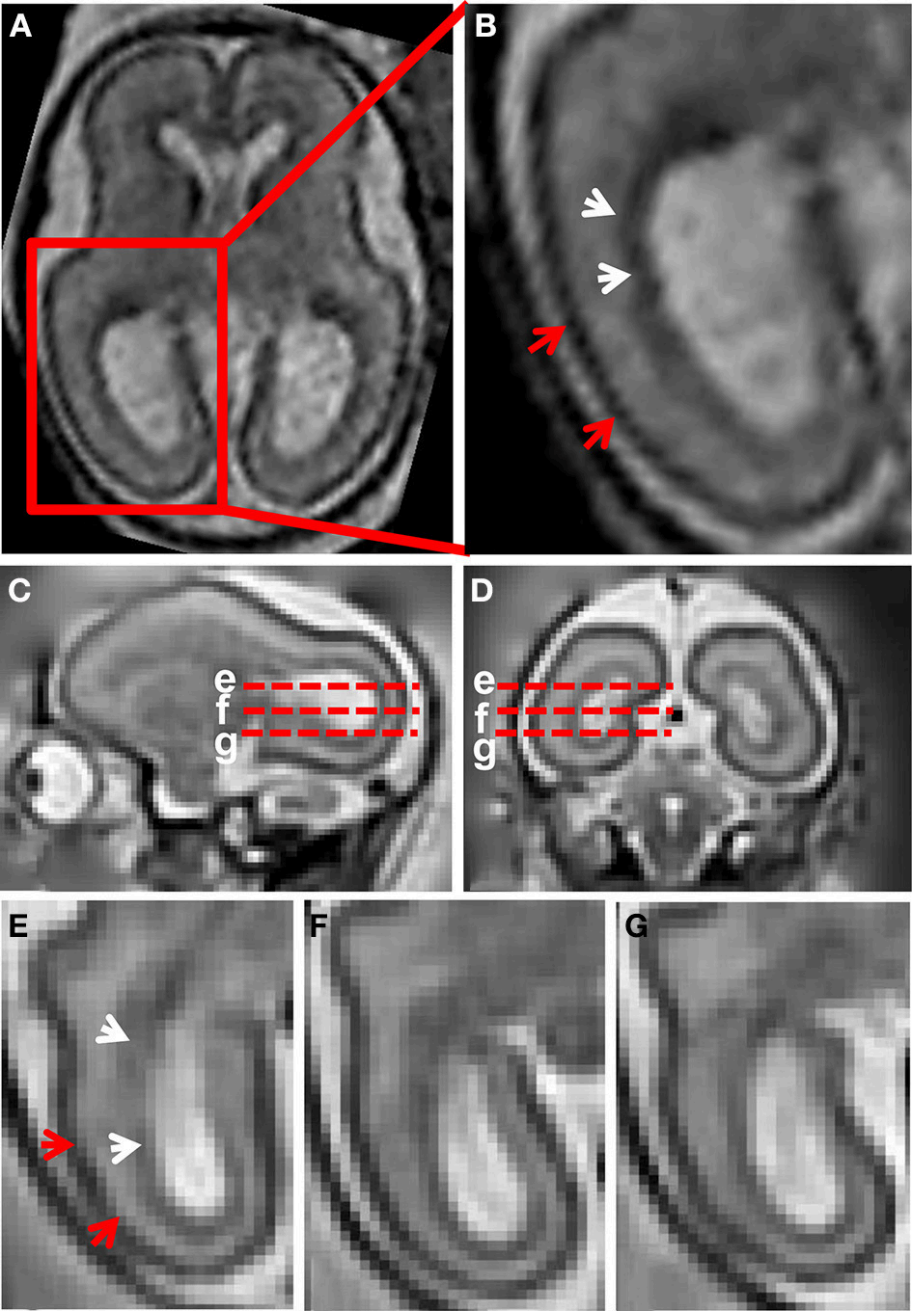
**T_2_-weighted ***in utero*** MRI of human and rhesus macaque fetal brain. (A)** T_2_-weighted axial image of a human fetal brain at 23 weeks gestation. In **(B)**, an enlarged view of the occipital and parietal region is shown. From the ventricular to pial surfaces, three tissue zones are apparent: germinal matrix (white arrows) is a dark zone proximal to the ventricular surface, the SP is a zone with higher intensity adjacent to the germinal matrix, and the CP (red arrows) is the superficial layer with the lowest intensity. For a G90 rhesus macaque fetus, T_2_-weighted images (0.5 mm isotropic) sagittal **(C)**, coronal **(D)**, and axial **(E–G)** views are shown. Axial views of the occipital region at the positions of the red dashed lines in **(C,D)** are shown in **(E–G)**. The three tissue zones apparent in human fetal MRI can also be observed in the fetal rhesus brain.

Figure 1A shows a T_2_-weighted axial image of a 23 weeks gestation human brain. An enlarged view of the caudal half of the brain, intersecting the occipital and parietal lobes, is shown in Figure 1B. At this stage of development, with standard image acquisition procedures, three tissue zones are apparent in the caudal telencephalon (Barkovich and Raybaud, 2012). The dark zone in T_2_-weighted images, proximal to the lateral ventricular surface, has been termed the germinal matrix (white arrows, Figure 1B). The zone with high image intensity adjacent to the germinal matrix corresponds to the SP. The CP is the most superficial zone, which is characterized by lower signal intensity than the SP in T_2_-weighted images (red arrow heads, Figure 1B).

**Figure 2:**
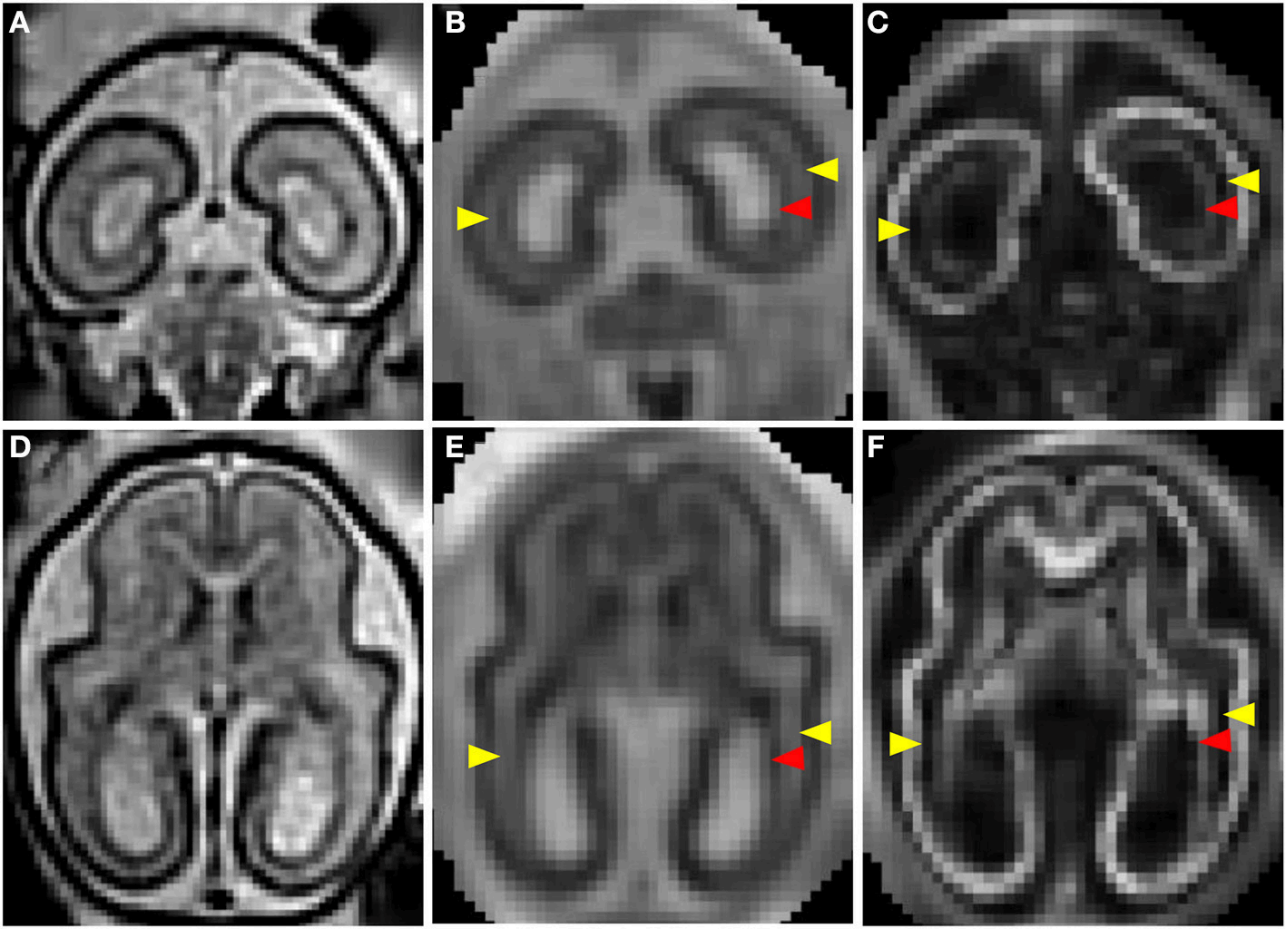
**T_2_- and diffusion-weighted ***in utero*** MRI of a G85 rhesus macaque**. Coronal and axial views of a T_2_-weighted image **(A,D)**, ADC map **(B,E)**, and FA **(C,F)** map. CP is characterized by relatively lower ADC **(B,E)** than the adjacent SP, and markedly high FA **(C,D)**. The lateral SP (yellow arrow heads) is sufficiently thick to be resolved from the CP and germinal matrix zones, exhibiting relatively high ADC **(B,E)** and negligible FA **(C,F)**. Within the germinal matrix (red arrow heads), the water ADC is relatively low **(B,E)**, and FA is higher than within the neighboring SP **(C,F)**.

Figures 1C–G shows a T_2_-weighted image acquired from a G90 rhesus macaque brain. Parasagittal and coronal views of high-resolution 3D reconstructed images, utilizing retrospective motion correction techniques, are shown in Figures 1C,D, respectively. Axial views of the caudal brain at the positions of the red dashed lines in Figures 1C,D are shown in Figures 1E–G. In spite of the overall smaller brain size, the same three tissue zones apparent in human fetal MRI were observed in high-resolution fetal images of the rhesus macaque brain. Diffusion MRI data, obtained from a fourth animal at a similar gestational age (G85), are shown in Figure 2. Coronal and axial views of a T_2_-weighted image, reconstructed at (0.5 mm)^3^ isotropic resolution, are shown in Figures 2A,D, respectively. As a consequence of the lower resolution of the acquired diffusion-weighted, compared to T_2_-weighted data, the ADC (Figures 2B,E) and FA (Figures 2C,F) maps were reconstructed at a lower resolution of (0.75 mm)^3^. In these images, the CP tissue zone is characterized by relatively lower ADC than the adjacent SP, and markedly high FA. The lateral SP is sufficiently thick to be resolved from the CP and germinal matrix zones, exhibiting relatively high ADC (Figures 2B,E, yellow arrow heads) and negligible FA (Figures 2C,F, yellow arrow heads). Within the germinal matrix, the water ADC is relatively low (Figure 2E, red arrow head), and FA is higher than within the neighboring SP (Figure 2F, red arrow head).

**Figure 3:**
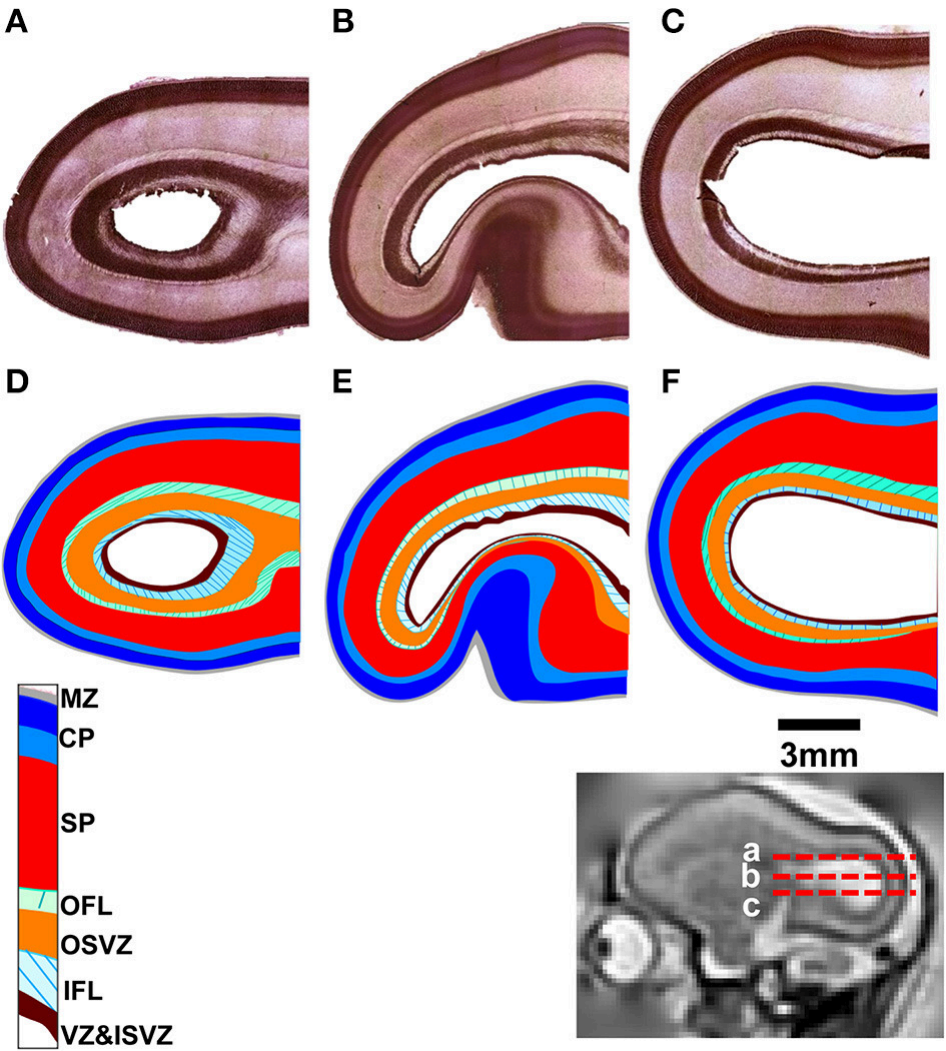
**Nissl-stained tissue for each fetal brain**. Horizontal sections are shown **(A–C)** at locations indicated by red dashed lines relative to a parasagittal T_2_-weighted image (inset). The tissue labeling scheme and color-code of Smart et al. (2002), delineating each tissue zone, is shown applied to each section **(D–F)**. MZ, marginal zone; CP, cortical plate; SP, subplate; OFL, outer fibrous layer; OSVZ, outer subventricular zone; IFL, inner fibrous layer; ISVZ, inner subventricular zone; VZ, ventricular zone.

Post mortem MRI and histological procedures were performed on the three G90 brains following *in utero* MRI. Figures 3A–C shows axial slices of Nissl-stained occipital and parietal lobes for each animal, revealing a more intricate laminar organization than the three tissue zone stratification supported by fetal T_2_-weighted MRI. The tissue labeling scheme, and the color-code delineating each zone of Smart et al. (2002) is adopted in Figures 3D–F, with the exception that the CP is separated into superficial (dark blue, Figures 3D–F) and deep (light blue, Figures 3D–F) components, following the intra-cortical Nissl staining intensity variation previously noted by others (e.g., Kostovic and Rakic, 1990). Based on the relative sizes and locations of tissue zones identifiable Figures 1–3, the CP observable by MRI corresponds to the marginal zone (MZ) and superficial and deep CP zones observable in Nissl-stained tissue. Thus, the SP and germinal matrix in MRI data overlap the remaining layers defined by Smart and co-workers. If the SP zones are coincident in MRI and histological images (an assumption investigated in more detail below), then the germinal matrix consists of five distinct tissue zones described by Smart and co-workers. These five zones extending from the ventricular surface outward toward the pial surface consist of the ventricular zone (VZ) and inner subventricular zone (ISVZ), the inner fibers layer (IFL), the outer subventricular zone (OSVZ), and the outer fibers layer (OFL; Figure 3).

**Figure 4:**
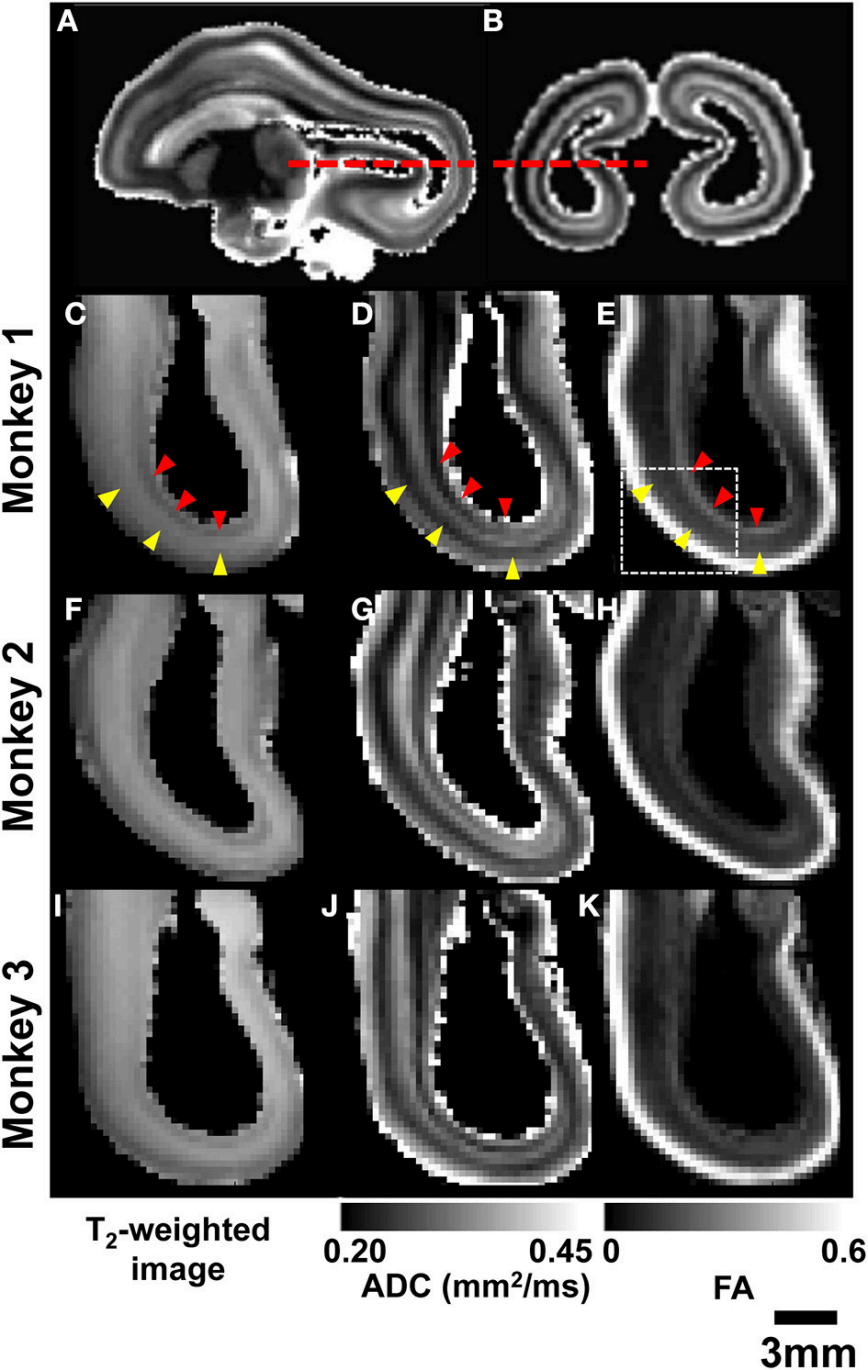
**Post-mortem MRI**. Parasagittal **(A)** and coronal **(B)** ADC maps obtained from monkey 1 are used to indicate the location of axial views of parietal and occipital lobes (red dashed lines). For all three G90 rhesus fetal brains, T_2_-weighted images **(C,F,I)**, ADC maps **(D,G,J)**, and FA maps **(E,H,K)** reveal consistent laminar patterns between the three brains. SP and germinal matrix zones indicated by yellow and red arrow heads, respectively.

Post mortem MRI data obtained from the three rhesus fetal brains shown in Figure 3 were examined to determine whether MRI-based contrast could be used to reveal greater detail in the primate brain laminar organization than the three heterogeneous zones idetentifiable in Figures 1, 2. Figures 4A,B shows parasagittal and coronal slices of ADC parameter maps from post mortem MRI performed on Monkey 1. Axial views of the occiptial and parietal telencephalon at the location indicated in Figures 4A,B (red dashed lines) are given for T_2_-weighted images, ADC maps, and FA maps of all three monkeys. As shown in Figures 4C,F,I, the three tissue zones observed *in utero* were also apparent in T_2_-weighted images of post mortem tissue. However, a consistent pattern of additional zones were observed in the high resolution ADC maps of all three post mortem brains, as shown in Figures 4D,G,J. Diffusion anisotropy maps in Figures 4E,H,K also revealed consistent patterns between brains, with high diffusion anisotropy in the CP, which is expected at this developmental stage (McKinstry et al., 2002). Notable diffusion anisotropy is also observed in periventricular zones (Figure 4E, red arrow heads).

**Figure 5:**
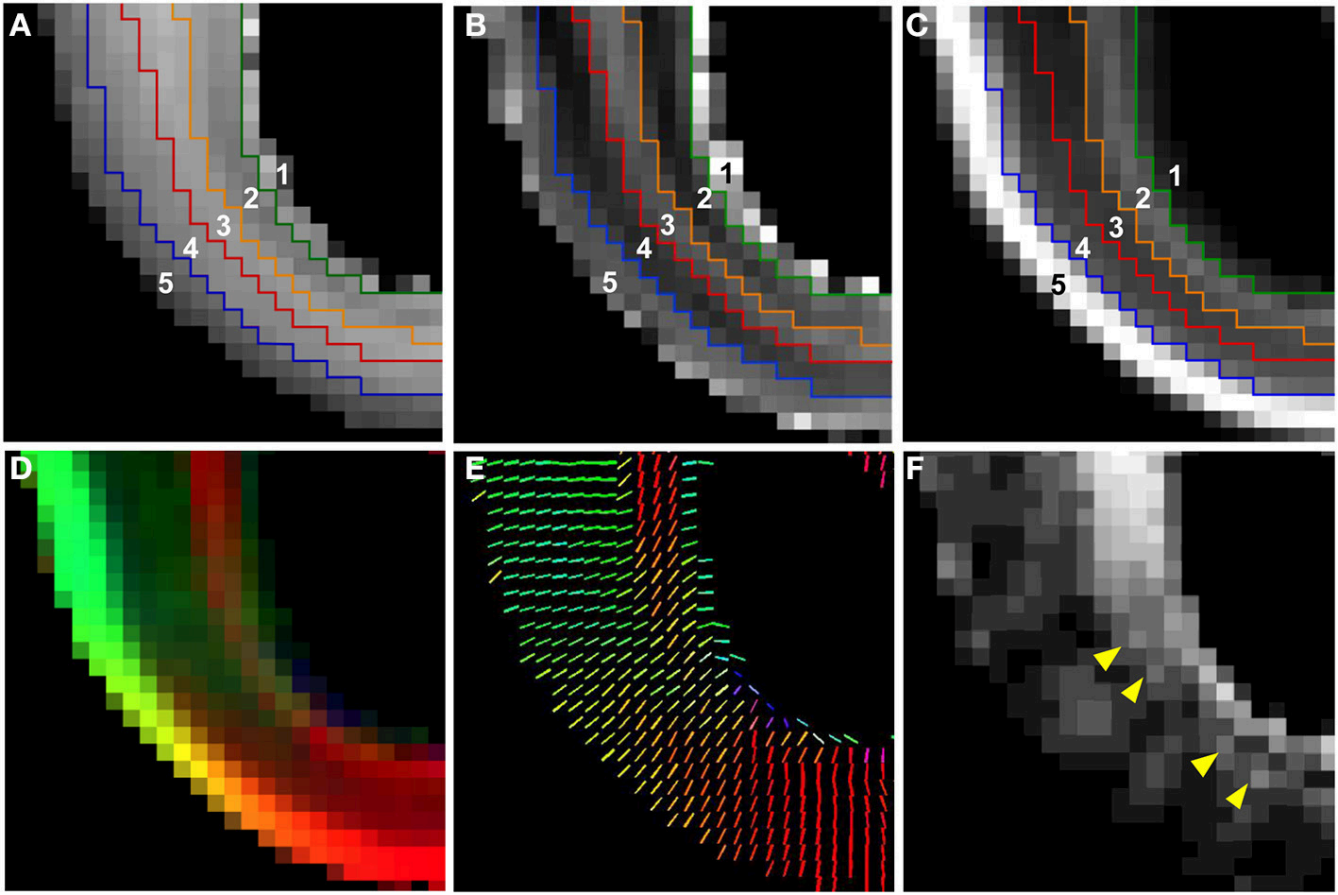
**Five tissue zones identified from post mortem MRI**. On an enlarged region of the axial views of Figure 4 (Figure 4E, white dashed box), five tissue zones were delineated on ADC maps **(B)** and the boundaries were projected onto T_2_-weighted image **(A)** and FA map **(C)** (see text for details). A diffusion anisotropy color map **(D)**, and a projection of the diffusion tensor primary eigenvector (V_1_) **(E)** is shown in for the same field of view. For color maps, red, green, and blue channels were scaled by the product of FA and the squared vertical, horizontal, and through-plane component of V_1_, respectively, with the FA map multiplied by a factor of 4. The angle θ between the radial direction vector, and the primary eigenvector of the diffusion tensor is also shown **(F)**. Yellow arrows in **(F)** indicate locations of local maxima in θ in the superficial part of Zone 2.

In Figure 5, an enlarged region of the axial views of Figure 4 (white box, Figure 4E) is shown. The ADC map was partitioned into five zones, and the boundaries between them were overlaid on the T_2_-weighted image (Figure 5A), the ADC map (Figure 5B), and the FA map (Figure 5C). Zone 1 is directly adjacent to the lateral ventricle, and consists of a 0.5–1 mm thick layer characterized by high water diffusivity (0.3–0.6 μm^2^/ms). Adjacent to this, Zone 2 is characterized by relatively lower water ADC (0.25–0.3 μm^2^/ms, between green and orange boundaries in Figures 5A–C). This zone exhibits increased FA (Figure 5C) and reduced T_2_-weighted image intensity (Figure 5A), relative to neighboring zones. Superficial to this, Zone 3 is characterized by relatively high water diffusivity (between orange and red boundaries in Figures 5A–C). Interestingly, there is a transition to an adjacent zone, Zone 4, of relatively lower water ADC (between red and blue boundaries in Figures 5A–C), that is not accompanied by a corresponding transition in FA or T_2_-weighted image intensity, and is not apparent in the lower-resolution ADC maps acquired *in vivo*. The fifth, and most superficial zone is characterized by relatively high water diffusivity, reduced T_2_-weighted image intensity, and extremely high water diffusion anisotropy.

**Figure 6:**
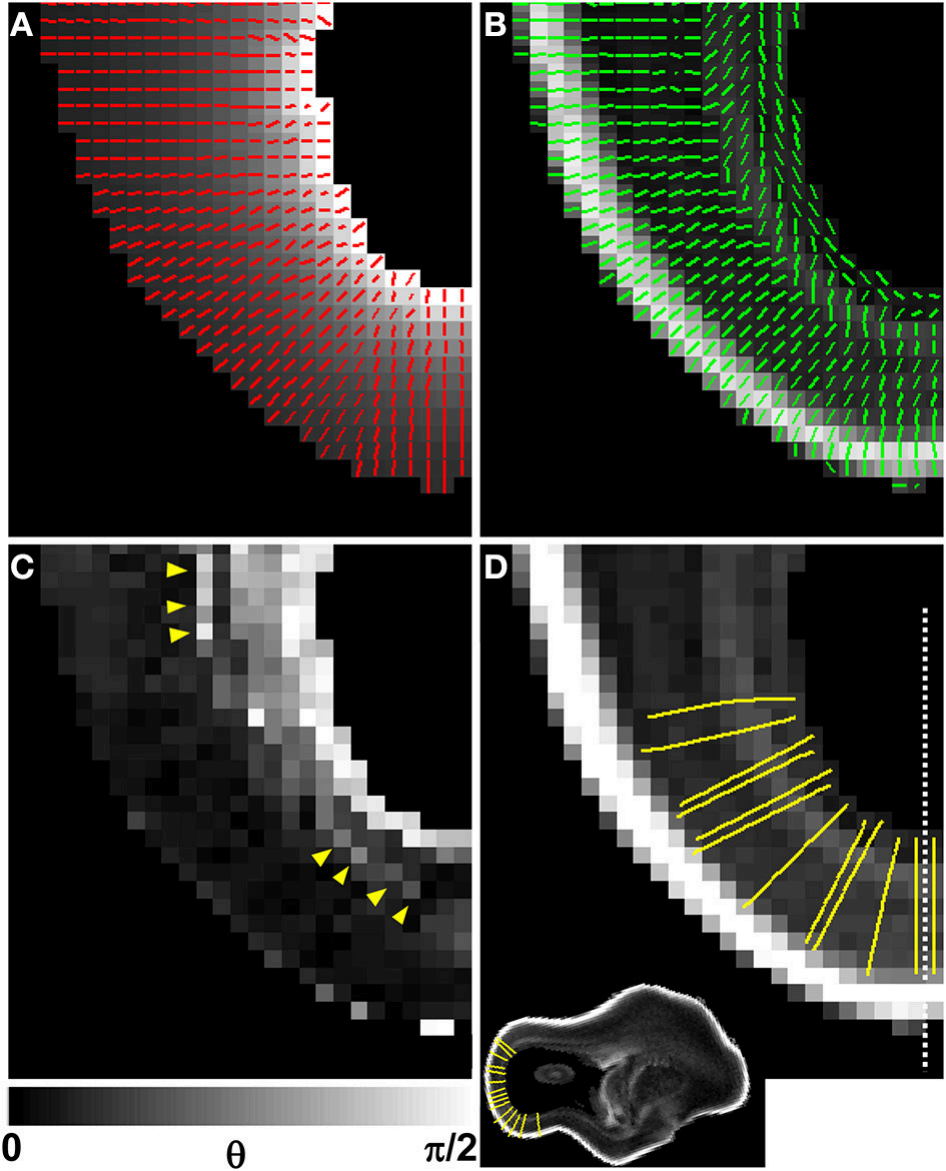
**Comparison of V_1_ to the local radial orientation**. The distance from the lateral ventricular surface was computed for each voxel in the occipital and parietal lobes (**A**, underlay), and displayed for the same animal as in Figures 4, 5, at a different axial plane. The 3D gradient of the distance matrix is oriented along the radial direction (**A**, red vectors). For the same field of view, the principal axis of the diffusion tensor for each voxel (green vectors) is overlaid on an FA map **(B)**. An image of angle θ between the radial direction vector, and the primary eigenvector of the diffusion tensor is shown in **(C)**. In general, θ is larger near the lateral ventricles, indicating water diffusion is least restricted in tangential directions, and it is smaller in more superficial lamina, indicating radially-oriented diffusion anisotropy. A local maximum in θ with respect to laminar position is observed near the ventricular surface (**C**, yellow arrow heads). Twelve radial streamlines are projected onto the same field of view in **(D)**. Each streamline originates at the center of a voxel bordering the ventricular surface, and ends at the pial surface. A parasagittal plane intersecting **(D)** at the dashed line location is shown as inset to provide a view that contains ventricular and pial radial streamline endpoints.

A diffusion anisotropy color map is shown in Figure 5D, and a projection of the diffusion tensor primary eigenvector field onto an axial plane is shown in Figure 5E. Particularly in lateral aspects of the brain, the diffusion tensor primary orientation differed dramatically between the subventricular zones and the CP. However, this arrangement was not found throughout the entire ventricular surface, as the periventricular diffusion tensor principal direction was oriented radially in the occipital pole in Figures 5D,E. In order to determine the radial direction at each voxel center, the distance from the lateral ventricular surface was computed for each voxel in the occipital and parietal lobes (Figure 6A underlay). The gradient of the distance matrix is oriented along the radial direction (Figure 6A, red vectors). For comparison, the principal axis of the diffusion tensor for each voxel in the same brain region is overlaid on an FA parameter map in Figure 6B. An image of the angle θ between the radial direction vector and the primary eigenvector of the diffusion tensor (Figure 6B, green vectors) is shown in Figure 6C. In general, θ is larger near the lateral ventricles, indicating water diffusion is least restricted in tangential directions, and it is smaller in more superficial lamina, indicating radially-oriented diffusion anisotropy. Superimposed on this trend, at many locations throughout the lateral and occipital cerebral wall, a local maximum in θ with respect to laminar position was observed near the ventricular surface (yellow arrow heads, Figure 6C). Comparisons of the radial vectors (Figure 6A) to the diffusion tensor primary eigenvectors (Figure 6B) confirms that voxels with high θ are characterized by non-parallel vectors in Figures 6A,B, in many cases as a result of a significant through-plane diffusion tensor primary eigenvector component. In Figure 5F, a θ map is displayed relative to the five-zone partitioning based on other MRI contrast mechanisms. Local maxima were observed in the superficial part of the second tissue zone at several locations within the lateral and occipital cerebral wall in this view as well (yellow arrows, Figure 5F).

**Figure 7:**
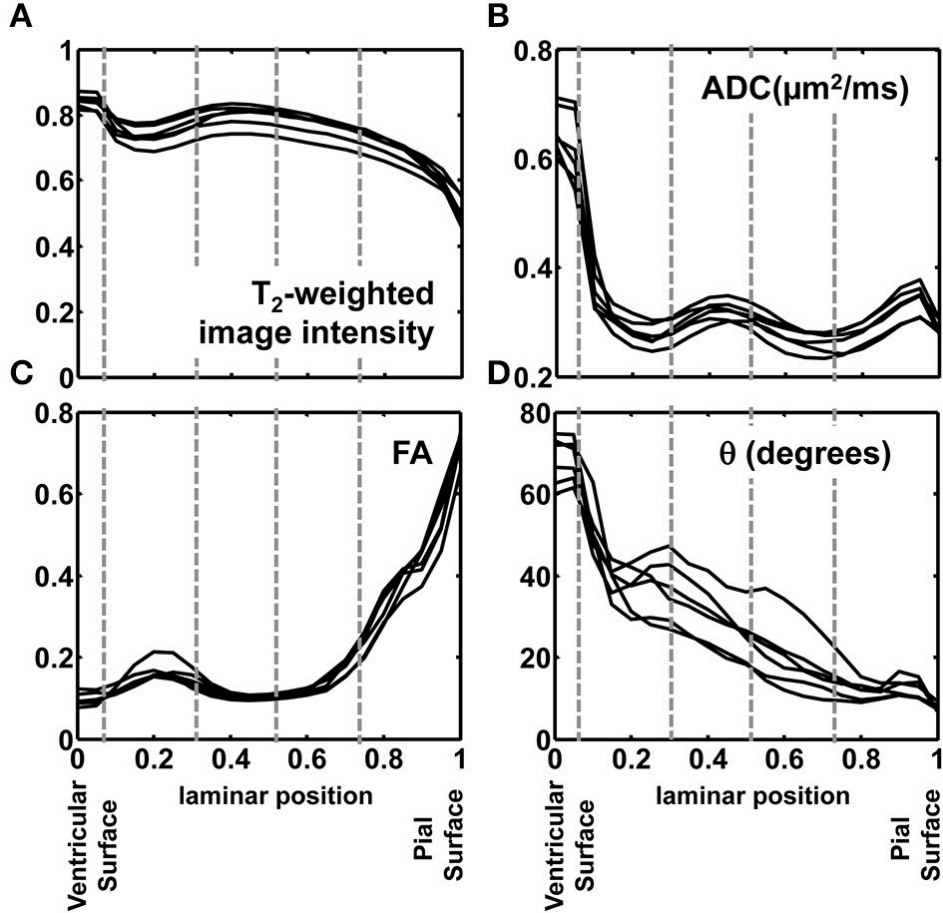
**Post mortem MRI parameters projected onto radial position**. T_2_-weighted image intensity, normalized to a common mean value **(A)**, ADC **(B)**, FA **(C)**, and θ **(D)** are plotted for each of the six hemispheres against relative position along the set of streamlines extending from the ventricular to the pial surface. The locations of borders between MRI-identified tissue zones are indicated as dashed vertical lines.

In order to quantitatively compare post mortem MRI data from the six hemispheres, each of the parameters presented in Figures 4, 5 were projected onto radial streamlines. As illustrated in Figure 6D, application of the streamline algorithm (using the “stream3” function of Matlab) to the radial vector field, using voxels bordering the lateral and caudal ventricular surface as seed points, yielded radial streamlines spanning the ventricular to pial surfaces. In Figure 6D, streamlines (yellow) are projected onto the FA map axial slice containing a set of seed points located at the ventricular surface. At this axial level, the radial streamlines have a significant component parallel to the dorsal/ventral axis, and therefore the 2D projection creates the appearance that they terminate prior to reaching the pial surface. A parasagittal inset is also shown for a set of streamlines in the occipital lobe, to demonstrate that they actually do extend to the pial surface. For each of the six hemispheres, an average of 600 radial streamlines were constructed, and the T_2_-weighted image intensity, ADC, FA, and θ were projected from each voxel intersected by each streamline. In Figure 7, the averaged parameters are plotted for each of the six hemispheres as a function of relative position along the set of streamlines extending from the ventricular to the pial surface. For T_2_-weighted image intensity, ADC, and FA, highly consistent locations and magnitudes of maxima and minima were observed. More variability between hemispheres was observed for θ. However, for all hemispheres, the rate of reduction in θ with laminar position was highest from 0.05 to 0.15, was very low from 0.15 to 0.35, and was moderate and uniform from 0.35 to 0.9, where it reached a minimal value of ~10°. The locations of borders between MRI-identified tissue zones are indicated as dashed vertical lines in the Figure 7 plots.

**Figure 8:**
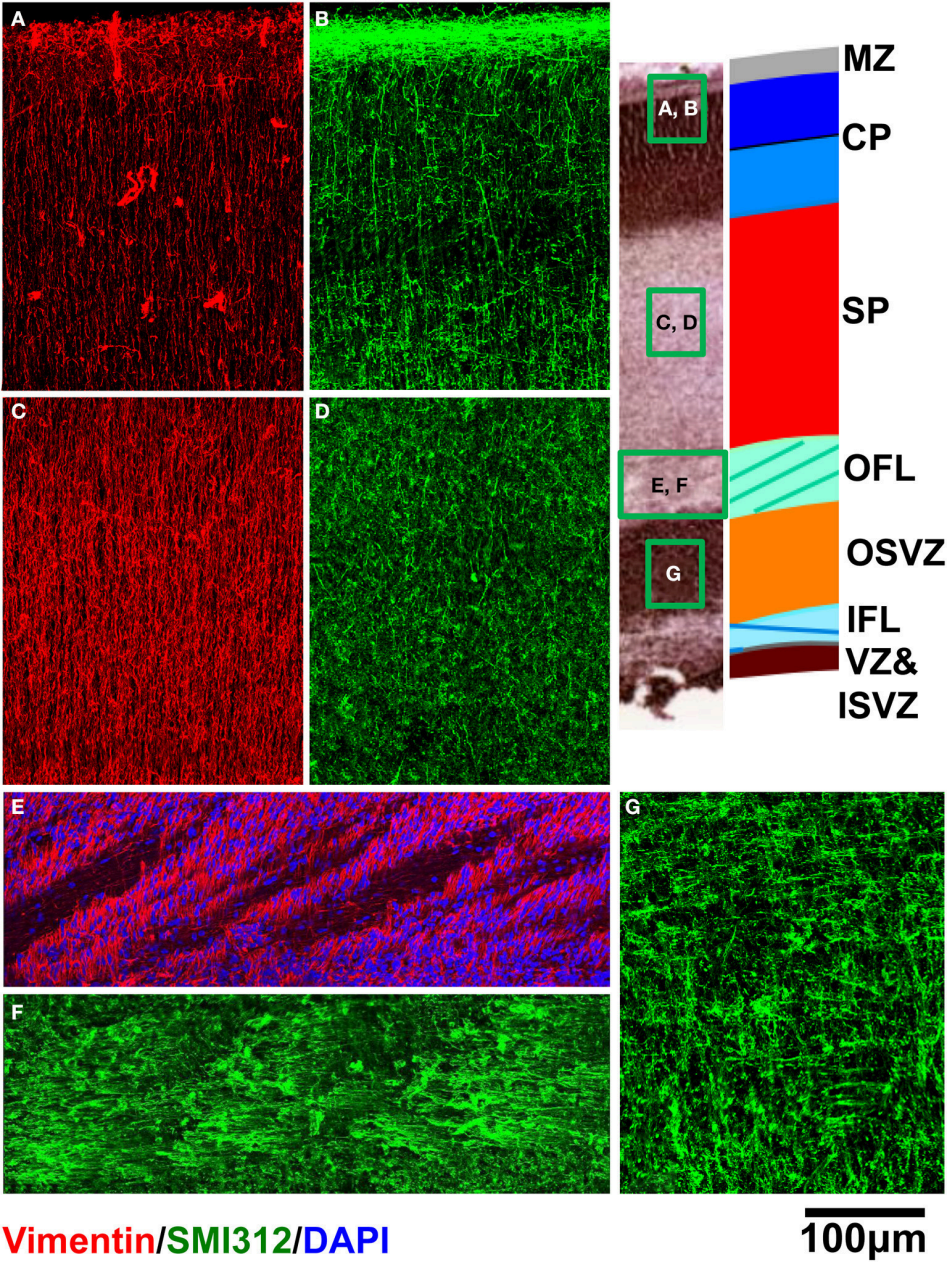
**Vimentin (red) and SMI312 (green) stained tissue sections from a representative rhesus macaque fetal brain are shown for regions of CP (A,B), SP (C,D), and OFL (E,F)**. DAPI (blue) is shown in **(E)**. For OSVZ, SMI312 staining is shown **(G)**.

Immunohistochemical analyses were performed to characterize the orientations of dominant cellular constituents across the cerebral wall. Within the CP (Figures 8A,B), SP (Figures 8C,D), and OFL (Figures 8E,F), radial glial processes revealed with vimentin staining appear consistently radially oriented, albeit in an interrupted manner within the OFL due to the presence of palisades of DAPI-negative fibrous tissue. Within the CP (Figure 8B), SMI312-positive axons are oriented radially. Within the OFL, in contrast, tangentially oriented axon fascicles are dominant structures (Figure 8F). A combination of diminished vimentin-positive, radially-oriented structures, and increased tangentially-oriented axon structures in the OFL, coincides with the laminar position of 0.25 in Figure 7. Interestingly, within the OSVZ, where inconsistent orientations of the diffusion tensor primary eigenvector are observed (Figures 5D,E), large compliments of radial as well as tangential axons are apparent (Figure 8G).

**Figure 9:**
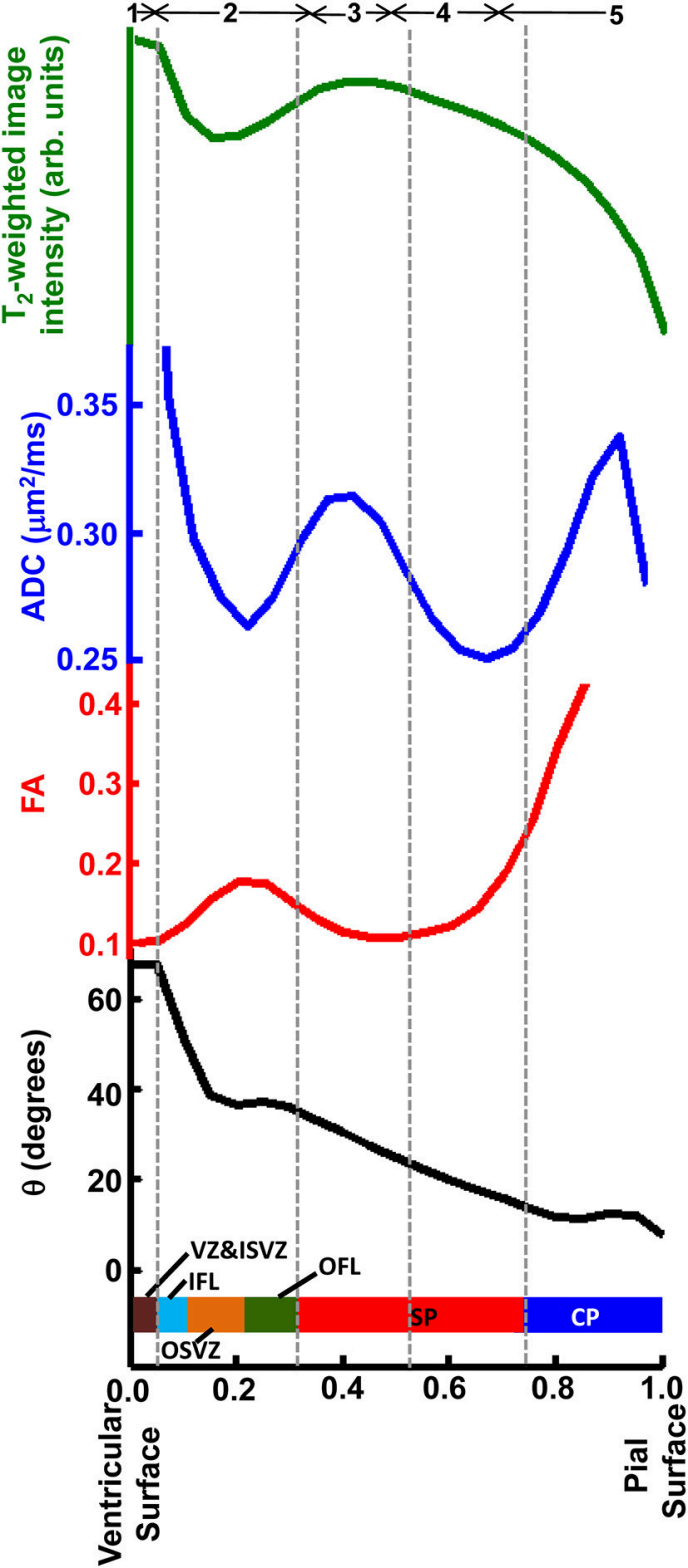
**Averaged T2-weighted image intensity (green), ADC (blue), FA (red), and θ (black) from all six hemispheres were plotted against relative laminar position**. Six tissue layers reported in Smart et al. (2002) were displayed relative to the abscissa and five tissue zones identified on post-mortem ADC maps, indicated by gray dashed lines.

In Figure 9, averages for the six hemispheres are plotted as a function of laminar position for the four parameters shown in Figure 7. The locations of maxima and minima for each parameter added constructively, indicating that the relative positions and thicknesses of the tissue zones are consistent among the set of hemispheres. This regularity facilitated comparisons to data from Nissl (Figure 3) and immunohistochemical (Figure 8) staining, as illustrated along the Figure 9 abscissa.

## Discussion

### Assignment of zones identified by post mortem MRI to established histological lamina

Based on multiple MRI contrast mechanisms, it was possible to identify five distinct laminar zones in the rhesus parietal and occipital lobes at 90 days gestational age. The correspondence between lamina identified by MRI and histological methods have been previously established for zones near the pia (Kostovic et al., 2002; Kroenke et al., 2006, 2007; Huang et al., 2009; Kolasinski et al., 2013; Xu et al., 2014). However, the associations between MRI and histological studies for zones near the ventricular surface have not been as systematically characterized. As shown in Figure 9, diffusion anisotropy in Zone 5 is oriented radially (manifested by low θ values), and diffusion anisotropy increases with proximity to the pial surface. This zone has been assigned to the CP and MZ (Kroenke et al., 2007; Huang et al., 2009). Tangential, rather than radial structures reside in the MZ (Figure 8B). However, the MZ is too thin to be resolved by MRI experiments, and therefore diffusion anisotropy throughout the superficial zone is oriented radially. In previous work, the presence of the MZ has been revealed through partial volume averaging to result in reduced diffusion anisotropy relative to neighboring voxels that overlap the CP (Kroenke et al., 2007).

The SP is approximately twice as thick as the combined thickness of the CP and MZ (Figure 2). Therefore, the entirety of Zone 4, and at least part of Zone 3 must contribute to the SP, and the SP contains sub-lamina characterized by low water diffusivity (Zone 4) and high water diffusivity (Zone 3). This intra-SP gradient in the water ADC has been noted previously by Huang et al. (2006), and inferred by Kostovic et al. (2002) in post mortem MRI studies of fetal human brains, and is apparent in post mortem ADC measurements of the G90 baboon (Kroenke et al., 2005). The histological analyses performed here did not reveal differences between superficial and deep SP that could underlie the variation of water diffusivity within this zone. Howerver, Kostovic (Kostovic et al., 2002) has proposed that the region of high diffusivity relates to the hygroscopic properties of SP extracellular matrix components. Although the biochemical mechanism responsible for the gradient in water diffusivity is not completely understood, the intra-SP ADC gradient is a robust phenomenon, as it has been observed by multiple research groups in multiple gyroencephalic species. It is plausibly related to the biological function of this zone by providing a molecular environment appropriate for the diffusion of growth and signaling factors as well as defasciculation of axons to promote innervation of the cerebral cortex (Kostovic et al., 2002).

The border between SP and OFL was assigned to a position in the MRI-identified tissue zones by referencing the direction of anisotropic diffusion relative to the radial direction. As shown in Figure 8F, the OFL contains a high density of tangentially-oriented SMI312 positive fibers compared to the neighboring SP and OSVZ (Figures 8D,G, respectively). This tangentially-oriented structure gives rise to a local maximum in θ in Zone 2 (yellow arrowheads, Figures 5F, 6C) near the border between Zones 2 and 3 (Figure 9). Thus, the OFL forms the outer-most part of Zone 2, and the OFL/SP border coincides with the border between Zones 2 and 3. Tangentially-oriented structures within the OFL (Figure 8F) and IFL give rise to moderately high FA values with tangentially oriented diffusion anisotropy (Figures 5C,F, respectively). Thus, the ISVZ/IFL border is coincident with the border of Zones 1 and 2. Zone 1 is characterized by negligible diffusion anisotropy (Figure 5B), which is appropriate for the “randomly organized cells” of the ISVZ (Smart et al., 2002) and VZ. However, the extremely high values for the water ADC and T_2_-weighted image intensity within this zone, particularly for the inner-most voxels, indicate that significant partial volume averaging with aqueous solution in the adjacent lateral ventricles influences MRI parameter values for at least a subset of voxels. Further, the combined thickness of ISVZ and VZ is expected to be on the order of a single 0.3 mm-sided voxel (Figure 3), which additionally suggests that partial volume averaging confounds interpretations of MRI parameters within this zone. In Figure 9, the MRI-identified tissue zones (dashed gray lines) are aligned to the lamina defined by Smart et al. (2002), relative to the MRI parameters investigated here.

### Relevance to fetal brain measurements performed *in utero*

Aside from differences in achievable image resolution, the T_2_-weighted image contrast pattern observed in the fetal rhesus brain with post mortem MRI was identical to the pattern observed *in utero*. In turn, similar T_2_-weighted image contrast patterns were observed in rhesus and human fetal brains with *in utero* MRI. Thus, it is anticipated that the associations described here between histologically-identified tissue zones and post mortem MRI can be extended to the context of human *in utero* fetal brain images.

For diffusion-based MRI contrast, the increased image resolution obtained in post mortem conditions enabled identification and characterization of tissue zones in a manner that was not possible with data collected from fetal brains *in utero*. Specifically, within the SP, two tissue zones were identified in ADC maps of post mortem tissue. These zones could not be resolved in images collected *in utero* (Figures 2B,E). A likely reason for this difference is that partial volume averaging of the CP and the most superficial SP zone gives rise to a single combined zone with a lower ADC than the remaining component of the SP. A second possibility is that biophysical differences between *in vivo* and *ex vivo* tissue give rise to the low ADC zone within the SP observed in post mortem MRI, but not *in utero*. High resolution *in utero* images will be necessary to discern between these two possibilities. A second difference between the *ex vivo* and *in vivo* data was the extent to which subventricular zones and associated fibrous layers could be characterized. Within the germinal matrix, voxels with modest FA values are observable in lateral brain regions, but these were too thin to be resolved in occipital regions (Figures 2C,F). With the improved image resolution obtainable with post mortem MRI, this zone could be identified in the occipital pole of FA maps. Further, in post mortem images, regions with varying directions of diffusion anisotropy could be resolved, which facilitated the assignment of the OFL to the germinal matrix zones traditionally identified in fetal brain MRI. These findings imply that future improvements in achievable image resolution in fetal diffusion MRI will be met with qualitative improvements in the ability to characterize SP, germinal zones, and fibrous layers of the fetal brain.

### Comparison to other labeling conventions

Several naming systems have been described for the mid-gestation primate parietal and occipital brain (Kostovic and Rakic, 1990; Altman and Bayer, 2002; Kostovic et al., 2002; Smart et al., 2002; Bayer and Altman, 2005; Huang et al., 2006, 2009; Bystron et al., 2008). Herein, tissue zones identifiable by MRI were referenced to the naming system of Smart et al. (2002). Based on the order from the ventricular to the pial surface, and matching tissue zones with similar descriptions, an approximate alignment of multiple other naming systems to the five MRI-identified tissues zones of this study is given in Table 1. It is important to emphasize that the alignment proposed in Table 1 is derived from caudal brain data at mid-gestation. Regional differences in laminar organization (Altman and Bayer, 2002; Martínez-Cerdeño et al., 2012), as well as differences in developmental stage (Smart et al., 2002; Cunningham et al., 2013; Wang et al., 2015), are critical factors that influence the characteristics of the tissue zones listed in Table 1. Notably, in studies of the human brain, Altman and Bayer (Altman and Bayer, 2002; Bayer and Altman, 2005) subdivide the region that corresponds to SP in other studies to a relatively thinner SP, and stratified transition fields (STF) 1 and 2, and potentially in addition, STF3c. Given that variation we observe in the water ADC within the SP (following the naming system of Smart et al.) is also observed by Huang et al. (2006) in human tissue, it is tempting to speculate that STF2 of Altman and Bayer (Altman and Bayer, 2002; Bayer and Altman, 2005) corresponds to Zone 3 of this study, and STF1 and SP of Altman and Bayer (Altman and Bayer, 2002; Bayer and Altman, 2005) corresponds to Zone 4. However, we were unable to identify STF1 and STF2 boundaries in our histological data, and therefore we do not currently have direct evidence to support this association.

**Table 1:** **Terminology for morphological zones in the mid-gestation occipital and parietal lobes from different studies**.

**Histology**				**MRI**			
Pial surface	Smart et al., 2002	Altman and Bayer, 2002; Bayer and Altman, 2005	Bystron et al., 2008	Kostovic et al., 2002	Huang et al., 2006, 2009	Barkovich and Raybaud, 2012	This study
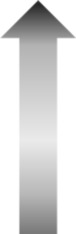	MZ	SGL	MZ	MZ	CP	CP	Zone 5
	CP	CP	CP	CP			
	SP	SP, STF1	SP	SP	SP	SP	Zone 4
		STF2					Zone 3
	OFL	STF3	IZ	IZ	IZ	GM	
	OSVZ	STF4,5	SVZ	SVCZ			Zone 2
	IFL	STF6		PVFZ			
	ISVZ	SVZ		VZ (GM)				
Ventriclular surface	VZ	NPE^a^	VZ				Zone 1

*^a^NPE, neuroepithelium; STF, stratified transitional field; SGL, subpial granular layer; IZ, intermediate zone; PVFZ, periventricular fiber rich zone; SVCZ, subventricular cellular zone. See Figure 1 for remaining abbreviations*.

### Varying cellular structures give rise to anisotropy in water diffusion

In white matter of the mature brain, myelinated axon fibers are the dominant structures contributing to water diffusion anisotropy. The situation in fetal brain is different, because there are multiple organized cellular constituents, in addition to axons, that influence water diffusion. Many cell structures in the developing brain are oriented radially (i.e., perpendicular to the ventricular and pial surfaces), such as radial glial cell processes throughout the cerebral wall (Figure 8; Xu et al., 2014), migrating neurons, and radially-oriented neural processes in the CP (Bock et al., 2010; Jespersen et al., 2012). Other cell processes, such as developing axon fibers, are oriented tangentially (parallel to the ventricular and pial surfaces). Developing axon fiber fascicles are most abundant in the IFL and OFL, wherein “palisades” of fascicles are interspersed with radial glial processes and associated migrating neurons (Smart et al., 2002). As a result of the dominant structural orientation changing with radial position, the direction of least restricted water diffusion, relative to the radial direction, varies across tissue zones (Kolasinski et al., 2013; Xu et al., 2014). It is therefore important to consider the effects of these additional cell structures when interpreting the results of diffusion anisotropy measurements in the developing brain. For example, application of diffusion tractography analysis procedures would be likely to generate fiber “tracts” that reflect properties of glial processes and neural dendrites, as well as axons, and as a result it would be erroneous to interpret the results of such analyses strictly in terms of axonal connectivity. Further, interpretations of changes in diffusion anisotropy with development, or differences in anisotropy between experimental groups, should similarly be interpreted with consideration of the various structures that have been shown to influence water diffusion in the fetal brain.

## Conclusion

Through the combined use of *in utero* MRI, post mortem MRI, and histological analyses of brains of rhesus macaques, the borders of previously-defined tissue zones have been assigned, with greater precision than previously possible in studies of primate brains, to transitions in various forms of MRI contrast. The *in vivo* and post mortem measurements performed on the same individuals demonstrate the close similarities in image contrast patterns under the two conditions and facilitate interpretations of *in vivo* data in terms of underlying cellular morphological features. Additionally, high resolution images obtained from post mortem tissue demonstrate that improvements in achievable *in utero* image resolution will enable the identification of tissue zone boundaries within individuals. Improved ADC map image resolution may additionally enable more complete characterization of sub-lamina with the SP.

### Conflict of interest statement

The authors declare that the research was conducted in the absence of any commercial or financial relationships that could be construed as a potential conflict of interest.
